# The expression and localization of a novel protein phosphatase inhibitor 2810408A11Rik in mouse testis and sperm

**DOI:** 10.1016/S1674-8301(12)60020-7

**Published:** 2012-03

**Authors:** Ye Bi, Mingxi Liu, Wenjiao Tu, Yibo Wu, Xuejiang Guo, Zuomin Zhou, Jiahao Sha

**Affiliations:** State Key Laboratory of Reproductive Medicine, Department of Histology and Embryology, Nanjing Medical University, Nanjing, Jiangsu 210029, China.

**Keywords:** 2810408A11Rik, testis specific, protein phosphatase inhibitor, capacitation

## Abstract

This study investigated the expression and distribution of 2810408A11Rik in mouse testis and sperm, and explored its role in spermatogenesis and sperm function. The expression levels of *2810408A11Rik* mRNA in multiple tissue samples were analyzed using bioinformatic resources and RT-PCR technique. A specific rabbit polyclonal antibody was prepared by prokaryotic expression of 2810408A11Rik recombinant protein and utilized for animal immunization. Western blotting, immunohistochemistry and immunofluorescence were used to detect the expression and distribution of 2810408A11Rik. The results of the bioinformatic analysis and RT-PCR showed that *2810408A11Rik* mRNA was specifically expressed in mouse testis, and 2810408A11Rik protein included a protein phosphatase inhibitor domain. Western blotting assays, immunohistochemistry and immunofluorescence confirmed the expression of 2810408A11Rik protein in mouse testis, especially in post-meiosis round and long spermatids, and that it is localized in the acrosome and the post-nucleus area of sperm. Our findings suggest that 2810408A11Rik may play an important role in spermatogenesis, sperm capacitation and fertilization.

## INTRODUCTION

Spermatogenesis is a continuous cellular process of division and differentiation. The ultimate goal is to maintain the stable transmission of paternal genetic information[1]-[5]. This cell differentiation process involves a complex series of morphological changes, including spermatogonia mitosis, spermtocyte meiosis to form spermatids, and then the development of round spermatids into sperm with normally structured heads, necks and tails. The spermatogenesis process is tightly regulated by many complex factors, including genetic program expression, regulation of specific molecules and genes, and the complex regulation of protein post-translational modifications. Protein post-translation modification plays a very important role in living organisms; it makes protein structure more complex, the protein function more complete, the regulatory function more precise, and the protein role more specific[6]-[9]. Among protein post-translational modifications, phosphorylation mainly participates in cell-signal transduction, cell proliferation, cell growth and differentiation, and other physiological processes. In fact, most cell functions are regulated by reversible protein phosphorylation. Studies have indicated that at least 30% of total proteins could be modified by phosphorylation. During the process of phosphorylation modifications, cell morphology and function state may change[10]-[14].

Numerous studies have shown that protein post-translational phosphorylation modification plays important roles in spermatogenesis and subsequent sperm capacitation, acrosome reaction and fertilization processes. Protein phosphorylation modification participates in the entire differentiation process, from spermatogonia to spermatids, and it is also closely related to fertilization. The normal protein phosphorylation and dephosphorylation role could be reflected in the following areas: to ensure normal meiosis, DNA damage repair, normal intracellular signal transduction pathway activation and inactivation, the correct regulation of spatial and temporal gene expression, normal energy metabolism, normal formation of sperm heads, and the normal functioning of capacitation and acrosome reactions to ensure successful fertilization[15]-[19]. During these protein phosphorylation modification processes, protein phosphatases and their inhibitors, which regulate protein dephosphorylation, play an important role. In our laboratory, we studied the regulation of protein post-translational modification in the spermatogenesis process, especially the role of phosphorylation modification. We screened numerous proteins which participate in post-translational modification during spermatogenesis, including 2810408A11Rik. Bioinformatic analysis suggested that 2810408A11Rik is a novel protein phosphatase inhibitor. We predicted that it might have a role in regulating protein phosphorylation. To date, there have been no published research reports on the expression and function of this protein. This study analyzed the expression and localization of 2810408A11Rik in mouse testis and sperm, and explored its role in the spermatogenesis and fertilization processes.

## MATERIALS AND METHODS

### Sample collection

Male mice (10-days, 2-weeks, 3-weeks, 4-weeks, 5-weeks, and 3-6 months old) were sacrificed to obtain tissue samples. Mature sperm was obtained from 3-6 month-old mice by making small incisions throughout the cauda epididymis followed by extrusion and suspension in phosphate-buffered saline (PBS) or culture medium [human tubal fluid [(HTF) media, In Vitro Care, Frederick, MD, USA]. To evaluate sperm capacitation, sperm was suspended in HTF medium and then cultured at 37°C for 100 min. Imprinting control region (ICR) white mice were maintained under a controlled environment of 20-22°C, with 12/12 h light/dark cycle, 50-70% humidity, and food and water ad libitum. Animal care and experimental procedures were conducted in accordance with the Animal Research Committee guidelines of Nanjing Medical University.

### Bioinformatics study

Pfam software (http://pfam.sanger.ac.uk/) was used to analyze the conserved domain in 2810408A11Rik. The expression level of 2810408A11Rik mRNA in various tissues was analyzed using GNF SymAtlas (http://biogps.gnf.org/#goto=welcome). The SymAtlas database covers the transcript expression level of different genes in various mouse tissues, based on Affy microarray.

### Reverse-transcription polymerase chain reaction (RT-PCR)

Multiple adult mice tissues, including the heart, liver, spleen, lungs, kidneys, brain, stomach, intestines, pancreas, skeletal muscle, epididymises, testes, uterus and ovaries, were collected and homogenized. Total mRNA was extracted according to the Trizol RNA isolation protocol (GibcoBRL, Grand Island, NY, USA) and reverse-transcribed into cDNA with AMV reverse transcriptase (Promega, Madison, WI, USA). The cDNA was PCR amplified according to the manufacturer's instructions using the following conditions: denaturation at 95°C for 30 s, annealing at 58°C for 30 s and extension at 72°C for 30 s; 35 cycles. Primers for 2810408A11Rik were: forward, 5′- GCGGATGAGCCCAGAACCCC-3′; reverse 5′- GCGGGAGCCGGACCTCAGAT-3′. The product size for *2810408A11Rik* was 554 bp. β-actin was used as internal control.

### Expression of recombinant proteins and antibody production

The full-length coding sequence of 2810408A11Rik was sub-cloned into the pET28a expression vector (GE Healthcare, San Francisco, CA, USA) encoding 6 *N*-terminally located histamine residues to obtain recombinant 2810408A11Rik protein. The construct was subsequently used to transform competent BL21 (DE3) pLysS cells. The transformed cells were grown in LB medium (10 g tryptone, 10 g yeast ex-tract, 5 g NaCl) containing kanamycin (50 µg/mL). When the cell concentration reached 1.7×10^8^ cells/mL, isopropyl-1-thio-β-D-galactopyranoside was added to a final concentration of 1 mmol/L to induce the expression of the 2810408A11Rik recombinant protein. After 6 h of induction at 37°C, cells were collected by centrifugation at 3,000 rpm and suspended in a buffer containing 8 mol/L urea. The cells were sonicated for 10 min on ice, and then centrifuged at 10,000 *g* at 4°C for 30 min. The recombinant protein in the supernatant was purified by HPLC (AKTA Basic, Amersham Biosciences, Santa Clara, CA, USA) under denaturing conditions according to the manufacturer's protocol (HiTrap™ Chelating HP 1 mL column) and the purified His-2810408A11Rik was refolded by dialysis against a decreasing linear gradient of denaturing buffer.

Antibody against 2810408A11Rik was produced by immunizing male New Zealand rabbits with purified recombinant 2810408A11Rik. The antisera titer was determined by ELISA. When the serum antibody titer reached 10^6^, the rabbits were sacrificed for serum collection.

### Protein extraction and immunoblot analysis

Testicular tissue and spermatozoa were homogenized (Ultra–Turrax^®^, IKA, Germany) and then treated with a lysis buffer containing 7 mol/L urea, 2 mol/L thiourea, 4% (*W/V*) 3-[(3-cholamidopropyl)-dimethylammonio]-1-propane sulfonate (Chaps), 2% (*W/V*) dithiothreitol (DTT) in the presence of 1% (*V/W*) protease inhibitor cocktail (Pierce Biotechnology, Rockford, IL, USA). The extracted protein concentration was determined with a Bio-Rad DC protein assay[20] kit (Bio-Rad Laboratories, Inc., Mississauga, ON, Canada) using bovine serum albumin (BSA) as the reference. The capacitating medium was collected after capacitation culture and ultrafiltrated (Amicon Ultra-15 centrifugal filter unit with Ultracel-50 membrane; Millipore, USA) to isolate proteins from the medium.

Protein extracted from the testis (60 µg/10 µL) and spermatozoa (50 µg/10 µL) were subjected to SDS-PAGE [12% (*W/V*) polyacrylamide gel] and the resolved proteins were transferred to a nitrocellulose membrane. The membranes were blocked with 1% skimmed milk in Tris buffer saline (TBS), and immunoblotted with the anti-2810408A11Rik serum, antigen pre-absorbed anti-2810408A11Rik serum, and a horseradish peroxidase-labelled secondary antibody(Beijing ZhongShan Biotechnology Co., Beijing, China). Immunoreactivity was detected using an enhanced chemiluminescence reaction (ECL) kit (Amersham Biosciences) and the images were captured by a FluorChem^®^ 5500 imaging system (Alpha Innotech, San Leandro, CA, USA). The molecular mass of the proteins were deduced by comparison with molecular mass standards (New England BioLabs, Ipswich, MA, USA).

### Immunohistology

Bouin's fixed testes were embedded in paraffin, sectioned at 5 µm, and mounted on silane-coated slides. For immunohistochemistry, sections were dewaxed and rehydrated through alcohol to distilled water, followed by incubation in 2% hydrogen peroxide to quench endogenous peroxidase activity and PBS wash. Subsequently, they were blocked with goat serum (Beijing ZhongShan Biotechnology) for 2 h and incubated with primary antibody (anti-2810408A11Rik rabbit serum; 1:2,000) overnight at 4°C. Following three washes in PBS, sections were incubated with horseradish peroxidase (HRP) conjugated goat anti-rabbit secondary antibody (Beijing ZhongShan Biotechnology) for 1 h at room temperature. Immunoreactive sites were visualized with diaminobezidine (DAB) and mounted for examination under bright field microscopy (Axioskop 2 plus, Zeiss, Germany). For negative control, anti-2810408A11Rik rabbit serum was preabsorbed with the 2810408A11Rik recombinant protein.

### Immunofluorescence

Mouse sperm samples, before-and-after capacitation culture, were fixed with 4% paraformaldehyde/PBS for 1 h, permeabilized with 0.2% Triton X-100 /PBS for 20 min at 37°C, and then blocked with goat serum (Beijing ZhongShan Biotechnology) for 2 h at room temperature. Following incubation with anti-2810408A11Rik serum overnight at 4°C, sperm were incubated with the secondary anti-rabbit IgG labeled with fluorescein isothiocyanate (FITC, Beijing ZhongShan Biotechnology) for 1 h at room temperature and observed under a ZEISS Axioskop plus2 fluorescent microscope at an excitation wave of 470 nm. For negative controls, anti-2810408A11Rik rabbit serum was preabsorbed with the 2810408A11Rik recombinant protein.

## RESULTS

### Distribution of 2810408A11Rik transcript in various tissues in mouse

The amino acid sequence of 2810408A11Rik was obtained from the NCBI protein database and analyzed using the Pfam software to identify conserved domains. The result revealed the presence of a protein phosphatase inhibitor 2 (IPP-2) domain[21] (150-277 aa) in 2810408A11Rik.

**Fig. 1:**
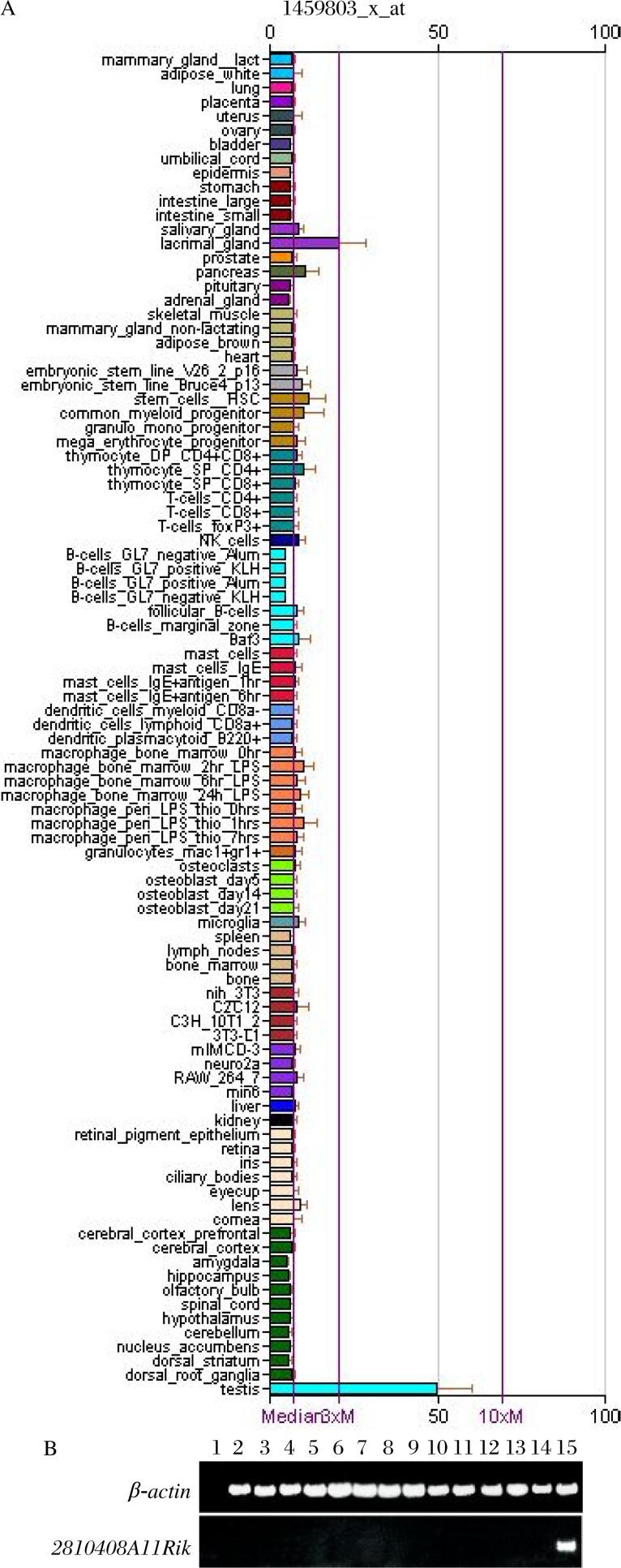
Testis-specific expression pattern of 2810408A11Rik in various mouse tissues and cells. A: Bioinformatics study using GNF Symatlas database showed that 2810408A11Rik mRNA was specifically and highly expressed in the testis. B: RT-PCR using RNA from various mouse tissues. Lane1: negative control; Lane 2: heart; Lane 3: liver; Lane 4: spleen; Lane 5: lung; Lane 6: kidney; Lane 7: brain; Lane 8: stomach; Lane 9: intestine; Lane 10: pancreas; Lane 11: skeletal muscle; Lane 12: uterus; Lane 13: ovary; Lane 14: epididymis; Lane 15: testis. β-actin was used as a loading control.

The mRNA expression level of 2810408A11Rik in ∼70 tissue and cell types was searched by submitting 2810408A11Rik to the GNF Symatlas database. The result showed that 2810408A11Rik mRNA was specifically and highly expressed in the testis (***Fig. 1A***). To confirm the testis specific expression pattern of 2810408A11Rik, we performed reserve-transcription polymerase chain reaction analysis with total RNA from different mouse tissues. The data showed that 2810408A11Rik was transcribed specifically in mouse testis and there was no transcript in any other tissues, including the heart, liver, spleen, lungs, kidneys, brain, stomach, intestines, pancreas, skeletal muscle, epididymises, uteruse and ovaries (***Fig. 1B***).

### Localization of 2810408A11Rik in mouse testes

The expression level of 2810408A11Rik in the testes was examined using immunoblot analysis. Compared with the recombinant protein pre-absorbed control, a specific band with an apparent molecular weight of approximately 47 kD was detected in testicular and spermatozoa protein extracts (data not shown) indicating that 2810408A11Rik is expressed in the testis and spermatozoa.

**Fig. 2:**
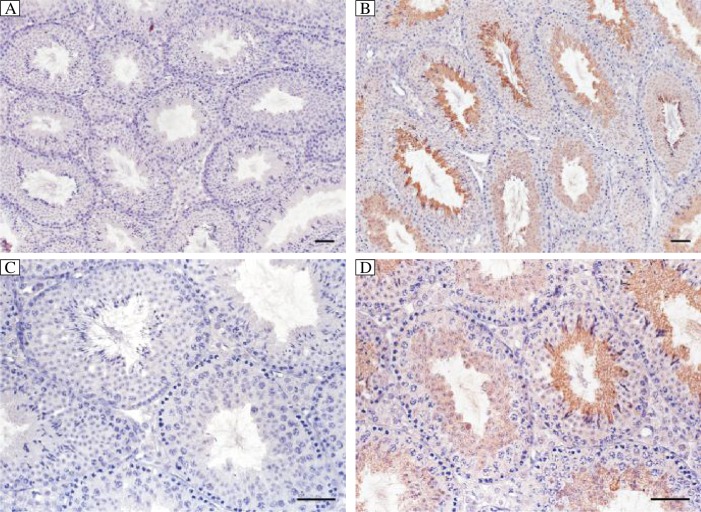
Expression and localization of 2810408A11Rik in adult mouse testis and spermatozoa. In mouse testis, 2810408A11Rik mainly localized in post-meiosis round and elongated spermatids (B, D) and the expression level increased as the spermatids underwent elongation. No positive signal could be seen in pre-absorbed control (A, C). Bar=50 µm.

**Fig. 3:**
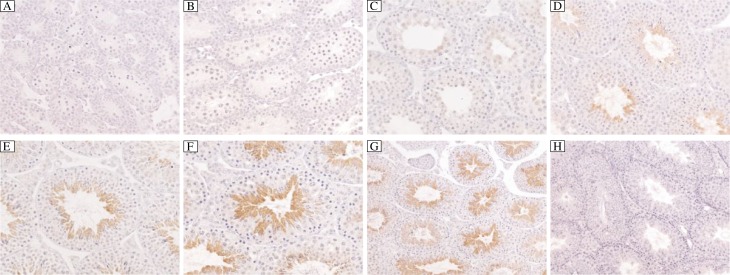
Localization of 2810408A11Rik in testis isolated from mice of different ages. 2810408A11Rik positive signals could only be detected after 3 weeks when the post-meiosis round spermatids cells first appeared in testis. The intensity of the signals increase increased as the round spermatids underwent the elongation process to form mature spermatozoa. A: testis from 10-day mouse; B: testis from 2-week mouse; C: testis from 3-week mouse; D: testis from 4-week mouse; E: testis from 5-week mouse; F-H: testis from 3-month adult mouse. A-F:×400; G,H:×200. A-G: Localization of 2810408A11Rik; H: pre-absorbed control.

Our immunohistochemistry study showed that positive signals were detected only in post-meiosis germ cells. The signals appeared at the round spermatid stage and increased significantly as the spermatids were elongated and mature spermatozoa were finally formed. This result was further confirmed by immunohistochemistry study in testes tissues from mice of different ages. The positive signals appeared only from the testis of 3 week old mouse, which synchronized with the time of the first appearance of round post-meiosis germ cells. No obvious signals were detected in any other cells in the testis. The negative controls (anti-2810408A11Rik serum preabsorbed with the 2810408A11Rik recombinant protein) produced only background levels of staining (***Fig. 2*** and ***Fig. 3***). These findings demonstrated that 2810408A11Rik was localized in mouse testes.

### Localization of 2810408A11Rik in mouse sperm

**Fig. 4:**
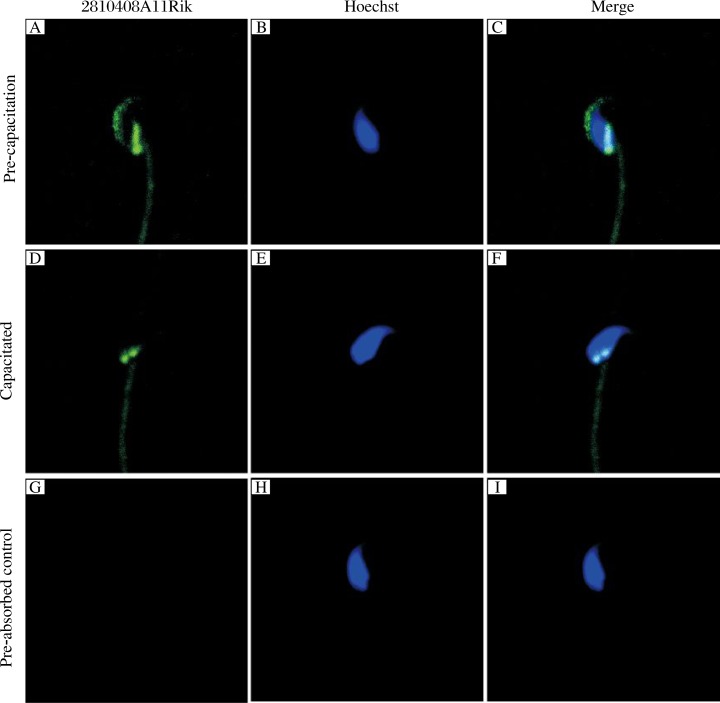
Localization of 2810408A11Rik in mouse sperm. 2810408A11Rik was localized in the acrosome and post-nucleus region in pre-capacitation sperm (A-C). But after capacitation (D-F), the signals in the sperm acrosome region were totally lost while the signal in post-nucleus region showed no significant change. No positive signals could be seen in pre-absorbed control (G-I). A, D, and G: 2810408A11Rik; B, E, and H: Hoechst; C, F, and I: Merge.

The localization of 2810408A11Rik in mouse sperm was further examined by immunostaining using the antiserum of the protein. Bright fluorescent staining was invariably observed in the acrosome and post-nucleus region of pre-capacitation sperm. As the sperm underwent capacitation, the positive signal in the acrosome became invisible while the signal in the post-nucleus was still as strong as that before capacitation. The negative controls (anti-2810408A11Rik serum pre-absorbed with the 2810408A11Rik recombinant protein) produced only background levels of staining (***Fig. 4***).

## DISCUSSION

Protein phosphorylation is an enzymatic reaction that transfers a phosphate group from one compound to another. It is the most common regulatory mode in organisms. Currently, there are many studies on protein phosphorylation. As one of the most important covalent modifications in organisms, the two reversible reactions, phosphorylation and dephosphorylation, are co-regulated by protein kinases and phosphatases. Phosphatase catalyzes the hydrolysis reaction to promote the process of protein dephosphorylation. Protein phosphatase inhibitors can inhibit the dephosphorylation reaction to maintain the phosphorylation state of the protein. The status and level of protein phosphorylation play a very important role in intracellular signal transduction, which can continuously respond to extracellular signals to make specific cascade amplified responses in order to maintain the normal organism functions by regulating protein activity and functional status.

In the process of spermatogenesis, protein phosphorylation participates in many cellular processes including meiosis, cell cycle regulation, gene transcription regulation, cell energy metabolism, and DNA damage reparation. It has also been shown that protein phosphorylation plays a key role in sperm capacitation and subsequent fertilization. Bioinformatics analysis predicted that 2810408A11Rik is a novel protein phosphatase inhibitor, but there has been no clear published data on the expression and function of 2810408A11Rik to date. In this study, we investigated the 2810408A11Rik protein as a potential novel protein phosphatase inhibitor.

Both the bioinformatics study and multi-tissue RT-PCR results showed that 2810408A11Rik exhibits a testis-specific expression pattern. Using Western blot with a highly specific polyclonal antibody, we showed abundant expression of 2810408A11Rik protein in adult mouse testis. Immunohistochemistry results showed that 2810408A11Rik protein is mainly located in post-meiosis round and elongated spermatids, and the expression amount increased as the sperm underwent maturation. These results suggest 2810408A11Rik plays its role in the sperm elongated maturation process by regulating protein phosphorylation status.

Changes in protein phosphorylation status and levels play a very important role in the process of sperm capacitation. Among these changes, the state of protein tyrosine phosphorylation in sperm is monitored as an indicator of sperm capacitation state, while the normal capacitation of the sperm is a key step to ensure normal fertilization. In our study, immunofluorescence studies of pre-capacitation and capacitated sperm showed that 2810408A11Rik is the located in acrosome and post-nucleus regions in pre-capacitated sperm while after capacitation, fluorescence in the acrosome region became significantly decreased or totally disappeared. In contrast, fluorescence in the post-nucleus region showed no changes. The localization and the change trends during capacitation in sperm acrosome region of 2810408A11Rik suggested it may play a role in the process of capacitation and acrosome reaction. 2810408A11Rik may regulate sperm capacitation and acrosome reaction through regulating the level of protein phosphorylation during the capacitation process. In addition, 2810408A11Rik located in the post-nucleus region may participate in the subsequent fertilization process, as this post-nucleus region had been recognized as the major fusion site of sperm-egg membrane. 2810408A11Rik may take part in this process, thereby regulating the success of fertilization.

In summary, our study showed that 2810408A11Rik exhibits a testis-specific expression pattern and it may act as a novel protein phosphatase inhibitor. Due to the specific localization of 2810408A11Rik in mouse testis, it may participate in the spermatogenesis process by regulating the level of protein phosphorylation, especially in post-meiosis round and elongated spermatids. In mature sperm, 2810408A11Rik may not only be involved in the process of sperm capacitation and acrosome reaction, but also in the process of sperm-egg membrane fusion and other biological events which ensure the success of normal fertilization. The potential roles of 2810408A11Rik require further investigation. To further clarify the function of 2810408A11Rik, research is needed to study how 2810408A11Rik enacts its role as protein phosphatase inhibitor, as well as identifying the protein phosphatase inhibited by 2810408A11Rik and its downstream proteins, whose activity and function are subsequently regulated.
